# Postgraduate trainees as simulated patients in psychiatric training: Role players and interviewers perceptions

**DOI:** 10.4103/0019-5545.74311

**Published:** 2010

**Authors:** Santosh K. Chaturvedi, Prabha S. Chandra

**Affiliations:** Department of Psychiatry, National Institute of Mental Health and Neurosciences, NIMHANS, Bangalore, Karnataka, India

**Keywords:** Postgraduate training, psychiatry, role plays, simulated patients

## Abstract

**Background:**

Teaching skills to enhance competence in clinical settings need to have a focus on learning how to do. This paper describes the subjective experiences and feedback of trainees who participated in a teaching technique using postgraduate trainees as simulated patients.

**Materials and Methods:**

The Objective Structured Clinical Assessment and Feedback was employed for training using trainees as simulated patients and interviewers. This exercise is performed in front of consultants and peers who subsequently provide feedback about the content and process using a structured format. In order to assess the subjective experience of the interviewer and the role players they were requested to provide structured feedback on several aspects. The trainee role player provided feedback on comfort in playing the role, need for further inputs, satisfaction regarding role play, satisfaction with the interview, and the overall effect of the activity. The trainee interviewer gave feedback on his/her level of comfort performing in front of a peer group, being watched, and evaluated in a group.

**Results:**

The feedback forms from 15 sessions were analyzed. Only two of the role players indicated that they felt very uncomfortable while the rest reported comfort. Twelve of the 15 trainees who simulated patients felt they needed more inputs to improve the clarity of the role play; however they all reported feeling satisfied with the role play or interview. The feedback from the interviewers indicated that most were comfortable in all aspects, i.e. conducting the interview, performing in front of a group, being evaluated, and given feedback in front of a group.

**Conclusion:**

The trainees report indicates that those simulating patients need more clarity on their roles and majority had no discomfort performing in front of a group. Interviewers were satisfied and comfortable with all aspects. On the whole, simulated interviews and role plays were found to be an acceptable teaching method by postgraduate psychiatry trainees.

## INTRODUCTION

It is known that “What you hear you forget, what you see you remember, and what you do, you understand” [Chinese Proverb]. On similar principles, the Millers’s triangle of learning principles has four stages of development–“knows, knows how, shows how and does” indicating steps from acquiring knowledge to performing a task in practice[1] and focus on competence-based learning and medical training.[2] Teaching skills to enhance competence in clinical settings need to have a focus on learning “how to do.” This paper describes the use of certain techniques to increase skill-based competence and also describes findings of a brief study on the perception of trainees involved in this activity. The paper concludes with a description of the advantages and disadvantages of such methods.

### OSCE as a training method

Assessment of competence can be done by objective structured clinical examination (OSCE), which has been used for examination of undergraduates and postgraduate medical students. The nature of conducting OSCE is such that it has a potential for use also as a teaching method to enhance competence-based training.[2] The OSCE format can be used in the training of communication skills, which are an essential element to the doctor patient relationship. The interaction can provide feedback on key interviewing skills such as paraphrasing, summarizing, and empathy. OSCE can also be used to teach history taking or for eliciting specific signs and symptoms.[2] While much of the literature has focused on the use of OSCE as a formative examination for students’ clinical skills,[3] not much has been discussed about its role in training.

### Simulated clinical interviews and role plays

Role plays and simulated interviews are also used for teaching clinical skills. Simulated and/or standardized patients have been used in medical education both in undergraduate and postgraduate settings. Role plays are a popular way of teaching communication skills to professionals. The first known effective use of simulated patient has been credited to Burrows and Abrahamson[4] who used them to appraise students’ performance in clinical neurological examinations.[2] Standardized patients are real (recovered) or simulated patients who have been coached to present a clinical problem.[5] Simulated patients can be trained actors playing a part of the patient.[6] Volunteer simulators who are not from acting profession have also been used.[7,8] Simulated patients have also been used from allied health programs, clinics in family medicine, and volunteer health care programs.[9] Thus simulated patients are not a homogeneous group and their only common characteristic is that of simulating real patients.

The term ‘standardized patient’ is increasingly used to indicate that the person has been trained to play the role of the patient consistently and according to specific criteria. The terms standardized patients and simulated patients are sometime used interchangeably. However, the simulated patient as defined by Barrows[10] is “a normal person who has been carefully coached to present the symptoms and signs of an actual patient.” Standardized patients, in contrast, are “people with or without actual disease who have been trained to portray a medical case in a consistent fashion. These people may portray their own problem(s) or ones based on those of other patients”.[11] The term ‘standardized patient’ is a broader term which covers both real and simulated patients. It does not indicate whether the patient being dealt with or discussed is a real or simulated one. Interviews with simulated patients are considered as useful as those with real patients, and have the advantage of not compromising on certain ethical issues. In a study[6] on clinical skills assessments using a sample of ten residents and comparing their interviews with four real patients and four simulated patients who were trained to present the same problem, no significant differences were noted in the number of questions asked in the history taking, physical examination, diagnosis or investigations. A recent review of the literature[11] concluded that simulated and standardized patients are useful and appropriate for teaching and assessment and are well accepted at both undergraduate and post-graduate levels. The review suggests an important place for role plays, as well.[11]

### Use of peer feedback of simulated interview as a training method

While supervisor feedback of simulated interviews has been considered an important part of training, not much is known about the value and acceptance of peer feedback. There is scope for two ways training when simulation happens in front of a peer group. On the one hand, peers observe the interview and learn to give systematic feedback and through that imbibe important skills, and simultaneously, the trainee gets feedback from the peers. The trainee doing the simulation/role play also learns important lessons, by way of being in the patient’s shoes for that period. The trainee who simulates the patient also is able to give relevant and important feedback to the interviewer. This way, a 15 min task helps in training several trainees at once, with different perspectives.

### The study

Role plays, simulated interviews, and an adaptation of the OSCE for teaching- objective structured clinical assessment and feedback (OSCAF) have been used as a teaching method at our centre over the last few years. The experiences and feedback given by the role players and interviewers are presented here. The adaptation of the OSCE method (OSCAF) for training of postgraduates in psychiatry and initial findings of trainee performance have been reported elsewhere.[12]

## MATERIALS AND METHODS

As part of the academic program, selected OSCAFSs are conducted on a weekly basis. The Unit has faculty members-three consultant psychiatrists, two psychiatry senior residents, four other consultants [psychologist, psychiatric social workers], six junior residents, and four or five other postgraduates. The trainees change every 3 months as a part of the rotation of training. An academic OSCAF roster is prepared for a 3 month rotation and the trainees know when they would be interviewing and the likely case scenarios. Prior to the exercise, the faculty decide what skills and knowledge need to be evaluated and devise a typical, culturally relevant case vignette for a particular topic. About an hour before the scheduled program, the case vignette is given to the trainee who is designated to perform the role play and simulate either the patient or the family member. [An example of a case description for assessment of sleep that is given to the trainee who simulates the patient-You are a 21 year old unmarried man/woman doing your postgraduate management course, who has become fearful of all vehicles following a bus accident four months ago and this fear has been affecting your social and occupational life. Your general physician referred you to the psychiatrist when you visited him for your sleep problems. The trainee is coached by the senior resident on how to simulate the patient in an authentic manner and adhering to the guidelines and description provided.

About 15 min before the OSCAF the interviewer is given the task that is to be carried out during the OSCAF. [For the above-mentioned vignette, a possible task for the interviewer may be as follows: A 21 year old man has been referred to you for sleep problems following a bus accident four months ago. Perform an assessment of his/her sleep problem.] During the interview, the interviewer and the interviewee role play the assigned task, while the faculty and trainees act as observers. The faculty members rate their feedback on a form devised for the purpose. One faculty member moderates the discussion and ensures that most trainees give systematic feedback to the interviewer with relevant examples of things done well or those that can be done better. These comments and feedback are given at the end of the 10 min interview.

To assess their subjective experience of the process, the interviewer and the role players are requested to fill different structured forms. The trainee performing the role play (of a patient or relative) provides a feed back on the following aspects-comfort in playing the role, need for further inputs for role play, satisfaction regarding role play, satisfaction with the interview, and the overall effect of the activity, positive or negative.

The trainee conducting the interview gives a feedback on his/her level of comfort as an interviewer, performing in front of a peer group, being watched and evaluated, with feedback in the presence of the group. The trainee is also asked to rate which aspect of the exercise was most useful for training and an overall satisfaction with his/her competence during the interview.

## RESULTS

The feedback forms from 15 such sessions were collected, compiled, and a one way frequency distribution was computed. The feedback from trainees doing the role play showed that only two felt very uncomfortable, seven felt a little comfortable, and other six rated themselves as being comfortable with the activity. Twelve of the 15 trainees felt they needed more inputs to improve the clarity of role play and none were dissatisfied with the role play or interview. Most (n=13) reported feeling ‘somewhat satisfied’ and two rated themselves as being fully satisfied. All but one reported a positive impact of the exercise, and one was not sure.

The feedback from the interviewers indicated that most were comfortable conducting the interview, performing in front of a group, being evaluated, and being given feedback in front of a group. The feedback by the consultants and other observers was reported to be the most helpful component of the exercise. Most (13/15) also reported being satisfied with the interview they performed.

## DISCUSSION

Simulated interviews and role plays in an OSCAF situation were found to be an acceptable and satisfying teaching method by psychiatry trainees. The structured feedbacks to the interviewers were found useful by the interviewer and role player. The comfort in performing role plays and satisfaction with role play and interview in front of peers and supervisors indicate that this teaching method can be used in postgraduate training. From the trainees feedback it appears that the group provides a safe space for learning new skills in the form of structured feedback from consultants and peers and majority of the trainees report feeling comfortable about this exercise. The use of simulation as a method of psychiatric education has been reviewed recently and the authors concluded that while the method enhances skills, it is not clear whether it translates into clinical practice.[13] Another study which used the same format as the current study evaluated the OSCE as a medical teaching method and reported low but significant correlations between the tutors‘ assessment and the students’ self-assessment and between the tutors’ assessment and the peer group’s assessment.[14] This congruence between observers in the assessments of role-played consultations using standard assessment criteria may be helpful for summarizing feedback to trainees.[14] This was not attempted in the current study, but is an interesting and important aspect to examine.

The academic activity was helpful in evaluating clinical skills in a simulated situation and as reported by other studies the multidisciplinary peer and consultant group fulfilled its objective in providing inputs and feedback from different professional perspectives (i.e. psychiatry, psychology, and psychiatric social work).[3,15] The trainees (interviewers) reported finding feedback to be the most beneficial aspect of the exercise. The finding that both groups of trainees (those who simulated the patient/caregiver and those who interviewed) found the exercise satisfying and comfortable while indicating acceptability do not necessarily indicate that this method is without its caveats.

### Advantages and disadvantages of OSCE as a training method

With our experience of adapting the OSCE as OSCAF for use as a training method we noted certain advantages and limitations, which are given below.

The disadvantages of this method are that ideal textbook scenarios may not be like real life situations and some scenarios may not allow assessment of complex skills. While this method might be expensive if trained actors are hired, it might be cost effective and a teaching exercise for the simulators if trainees are used for the simulation, such as we did.

However, based on the feedback from our trainees, it appears that adequate training is needed for those trainees who simulate patients or caregivers. This can be in the form of better and more elaborate descriptions of the role, rehearsal prior to the activity and also debriefing following the exercise particularly when simulating situations such as grief or suicide attempts. It will also be important to ensure that the trainee does not feel anxious or intimidated about performing in front of a group.

The advantages are that it simulates real life situations, is close to reality, is controlled and safe, and a feedback is available from the role player.[2] There is ready availability of the task when required, scenarios can be modified to the level of skills needed and scenarios can be easily acted upon. The trainee who simulates the patient or caregiver has an opportunity to experience how patients or relatives feel in an interaction. Whether this contributes to increase in empathy is a question worth studying.

The multiple feedback mechanisms inbuilt into this teaching exercise also appear to enhance satisfaction among the trainees as was evident in our study. The role players are also in an appropriate position to give more specific feedback to the interviewer about their experience like what questions upset them, confused them or were appropriate, what behaviors of the interviewer comforted them, and what behaviors upset them further. Such a feedback is difficult to get in real time clinical settings from real patients. Patients or their relatives would usually refrain from giving a critical or negative feedback to the interviewer who is responsible for their care. It would be interesting however to also get more systematic feedback from the peer observers to see how comfortable they feel with giving feedback to their peers.

Some books provide detailed methods of how to organize and conduct OSCEs and provide feedbacks. These also include instructions for the role players and interviewers,[16–19] which may be useful in training or for OSCE as an assessment of skills or competence.

There is not much literature on teaching methods used in postgraduate settings from India and there is a need for more research in the area given the limited number of teachers and complex cultural and social needs of patients presenting to psychiatry. There is also a need for documentation and research into different training methods both regarding their efficacy and acceptability.

## Appendix

OSCE feedback form: From the trainee doing role play

- Comfort in playing the role
  0 - Very uncomfortable
  1 - A little uncomfortable
  2 - Comfortable
- Clarity in playing the role-would have
  0 - Required all inputs
  1 - Require some inputs
  2 - No inputs required
- Satisfaction regarding role play
  0 - Not satisfied
  1 - Somewhat satisfied
  2 - Fully satisfied
- How do you rate the interview?
  0 - Not satisfied
  1 - Somewhat satisfied
  2 - Fully satisfied
- Overall feeling about Role play
  0- Negative
  1- Can’t say
  2- Positive
- Any measures to do it differently

OSCE feedback from Interviewer

1. Comfort
  - With self as interviewer
    0 - Very uncomfortable
    1 - A little uncomfortable
    2 - Comfortable
  - Performing in front of peer group
    0 - Very uncomfortable
    1 - A little uncomfortable
    2 - Comfortable
  - Being evaluated
    0 - Very uncomfortable
    1 - A little uncomfortable
    2 - Comfortable
  - Feedback in a group
    0 - Very uncomfortable
    1 - A little uncomfortable
    2 - Comfortable
2. Which component of today’s OSCE did you feel most helpful?
  - Interview
  - Role play
  - Improved communication
  - Feedback
3. Overall rating about interview
  0 - Not satisfied
  1 - Somewhat satisfied
  2 - Fully satisfied
4. Any measures to do it differently
